# Methods for Biomimetic Remineralization of Human Dentine: A Systematic Review

**DOI:** 10.3390/ijms16034615

**Published:** 2015-03-02

**Authors:** Chris Ying Cao, May Lei Mei, Quan-Li Li, Edward Chin Man Lo, Chun Hung Chu

**Affiliations:** 1 Faculty of Dentistry, The University of Hong Kong, Hong Kong, China; E-Mails: caoying0713@gmail.com (C.Y.C.); modelbaby1981@gmail.com (M.L.M.); hrdplcm@hku.hk (E.C.M.L.); 2 Key Laboratory of Oral Diseases Research of Anhui Province, Stomatological Hospital & College, Anhui Medical University, Hefei 230032, China; E-Mail: ql-li@126.com

**Keywords:** biomimetic, remineralization, dentine, non-collagenous proteins

## Abstract

This study aimed to review the laboratory methods on biomimetic remineralization of demineralized human dentine. A systematic search of the publications in the PubMed, TRIP, and Web of Science databases was performed. Titles and abstracts of initially identified publications were screened. Clinical trials, reviews, non-English articles, resin-dentine interface studies, hybrid layer studies, hybrid scaffolds studies, and irrelevant studies were excluded. The remaining papers were retrieved with full texts. Manual screening was conducted on the bibliographies of remaining papers to identify relevant articles. A total of 716 studies were found, and 690 were excluded after initial screening. Two articles were identified from the bibliographies of the remaining papers. After retrieving the full text, 23 were included in this systematic review. Sixteen studies used analogues to mimic the functions of non-collagenous proteins in biomineralization of dentine, and four studies used bioactive materials to induce apatite formation on demineralized dentine surface. One study used zinc as a bioactive element, one study used polydopamine, and another study constructed an agarose hydrogel system for biomimetic mineralization of dentine. Many studies reported success in biomimetic mineralization of dentine, including the use of non-collagenous protein analogues, bioactive materials, or elements and agarose hydrogel system.

## 1. Introduction

Dentine is a collagenous mineralized tissue, which contains 70% carbonated apatite, 20% organic matrix, and 10% water (by weight) [1]. Although collagen fibrils are the major organic component of dentine, non-collagenous proteins (NCPs) and glycoproteins (less than 10% of total organic content) play very important roles in the regulation of mineralization [2]. Demineralization and remineralization processes coexist in teeth during the entire life of an individual [3]. Dental caries is a dynamic disease process caused by an imbalance between demineralization and remineralization [4]. In pathological conditions, demineralization outweighs remineralization. Initial carious lesions affect the mineral phase of dentine and expose the collagen fibers, creating the conditions for a fast destruction of the entire dentine network, resulting in the degradation of collagen fibrils and a decrease in the mechanical properties of dentine [5]. Nowadays, dental fillings, such as amalgam or resin composite, have been used to repair dental caries. However, second caries often happen at the interface between the restorative materials and dentine. Tooth fillings need to be replaced due to being worn, cracked, or fractured. Thus, it is a major challenge for operative and preventive dentistry to induce the remineralization of hypomineralized carious dentine. The remineralization of demineralized dentine is the process of restoring minerals through the formation of inorganic mineral-like materials.

Dentine remineralization is more complex and less effective than enamel remineralization because there are residual seed mineral crystals on enamel, but these are absent in dentine lesions. Different strategies have been investigated to study the remineralization of dentine, such as fluoride and amorphous calcium phosphate-releasing resin [6,7]. Fan, *et al.* [8] reported that under the same remineralizing condition, remineralization occurred on the surface of acid-etched enamel but not on the surface of acid-etched dentine. This difference could be attributed to the fewer amounts of residual mineral crystals and the exposure of organic matrix (mainly type I collagen) on the acid-etched dentine surface. Thus, the classical ion-based crystallization concept may not be applicable for remineralizing completely demineralized dentine [9]. In biology, a biomineralization process is an organic, matrix particle-mediated, non-classical crystallization pathway [10]. It is generally believed that extracellular matrix proteins play an important role in controlling apatite nucleation and growth in the dentine biomineralization process. Existence of transient amorphous calcium phosphate (ACP) nano-precursors has been identified in many forms of biomineralization [11]. Biomimetic remineralization, a methodology that imitates the natural process of mineralization, represents a different approach to this problem by attempting to backfill the demineralized dentine collagen with liquid-like ACP nano-precursor particles. This bottom-up remineralization strategy does not rely on seed crystallites and may be considered as a feasible method for remineralization of demineralized dentine. This paper is a systematic review of the different published methods that successfully achieved biomimetic remineralization of human dentine.

## 2. Results

**Figure 1:**
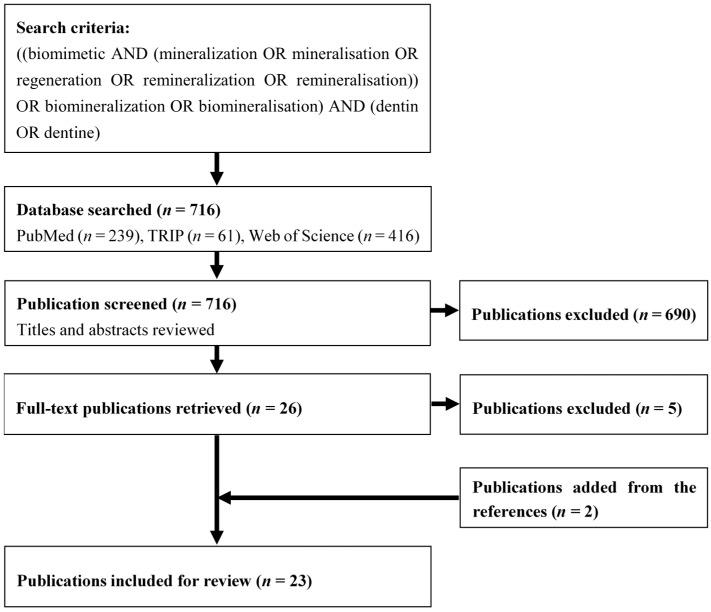
Flowchart of the search strategy.

The initial search identified 716 potentially eligible articles (239 articles in PubMed, 61 articles in TRIP, and 416 articles in Web of Science). After initial screening of titles and abstracts, 690 were excluded, leaving 26 articles for full-text analysis. After retrieving the full text, two articles were identified from the references of the included articles, and 23 were included in the systematic review (Figure 1). All 23 articles were *in vitro* studies published between 2004 and 2014.

The different biomimetic mineralization methods used in the included articles are summarized in Table 1. Sixteen studies used different analogues to mimic the functions of NCPs in biomineralization of dentine. Four studies used bioactive materials, which could release mineral ions to induce apatite formation on demineralized dentine surface [12,13,14,15]. One study used zinc as a bioactive element [16], one study used polydopamine [17], and another study constructed an agarose hydrogel system for biomimetic mineralization of dentine [18].

The dentine surface was demineralized (surface treatment) before biomimetic mineralization. Eleven studies used phosphoric acid (PA) as demineralization agent. Except for one study which used 20% PA [18], the concentration of PA reported was 32% to 37%. Six studies used ethylenediaminetetraacetic acid (EDTA) at 17% or 0.5 M concentrations [13,14,15,19,20,21]. In addition, three studies used guanidine chloride (GuCl) to remove the mineral-bonded NCPs after EDTA treatment. Preparation of artificial carious lesions by the pH-cycling procedure was reported in three studies [22,23,24]. The demineralizing solution containing calcium, phosphate, and acetic acid was used in 2 studies. Sodium hypochlorite was used to clean the dentine surface [12].

Different remineralizing mediums were used to provide calcium and phosphate ions. The most commonly used calcium-containing remineralizing medium was Portland cement, which acted as the calcium and hydroxyl ion-releasing source [14,15,19,22,23,24,25,26]. In addition, calcium phosphate remineralizing solution [17,27,28,29,30], casein phosphopeptide-amorphous calcium phosphate or CPP-ACP paste [31], artificial saliva [16,20,21,32], bioactive glass [12,13], calcium chloride solution [18,33], and metastable calcium phosphate solution [1] were also used. Regarding the phosphate-containing remineralizing medium, simulated body fluid (SBF) [12,19,22,23,24,26], phosphate-containing solution/gel [15,18,25,33], calcium phosphate remineralizing solution [1,17,27,28,29,30,31], artificial saliva [16,20,21,32], and phosphate-buffered saline (PBS) [14] were used in the included articles.

Some of the main findings of the studies were (i) hydroxyapatite crystals covered the dentine surface and occluded the dentinal tubules; (ii) dentine collagen fibrils were mineralized with evidence of interfibrillar and intrafibrillar remineralization; (iii) NCPs analogues facilitated the remineralization of dentine; (iv) bioactive glass facilitated the remineralization; (v) apatite was deposited within collagen fibrils; and (vi) dentine remineralization was achieved (Table 1).

**Table 1:** Summary of the *in vitro* studies on biomimetic mineralization on human dentine.

Authors, Year [Reference]	Method	Surface Treatment	Sources of Ca and P	Main Finding
Forsback *et al.* 2004 [12]	Bioactive glass	0.5% NaOCl (5 min)	Bioactive glass, SBF, Remineralization solution	Calcium phosphate layer covered the dentine surface
Vollenweider *et al.* 2007 [13]	Bioactive glass	17% EDTA (2 h)	Bioactive glass suspension	Bioactive glass facilitated remineralization
Tay *et al.* 2008 [25]	PAA, PVPA	37% PA (15 s)	Portland cement, PO_4_-containing fluid system	Interfibrillar and intrafibrillar remineralization of dentine
Reyes-Carmona *et al.* 2009 [14]	MTA, PBS	17% EDTA (3 min), 1% NaOCl (3 min)	MTA-PBS system	Apatite deposited within collagen fibrils
Gandolfi *et al.* 2011 [15]	Ca_2_SiO_4_ hybrid “smart” materials	17% EDTA (2 h)	Portland-derived mineral, CaAl_2_Si_2_O_8_, PO_4_ solution	Bone-like carbonated-apatite formed on dentine
Gu *et al.* 2011 [19]	PAA, PVPA	0.5 M EDTA, 4 M GuCl	Portland cement-based composite, SBF	Dentine remineralization with intrafibrillar mineral infiltration
Liu *et al.* 2011 [22]	STMP, PAA	pH-cycling	Portland cement, simulated body fluid system	STMP is a promising method to remineralize artificial carious lesion
Liu *et al.* 2011 [23]	PAA, PVPA	pH-cycling	Portland cement, biomimetic analogue-containing SBF	Intra and extrafibrillar mineralisation of collagen fibrils
Gu *et al.* 2011 [26]	STMP, PAA	32% PA gel (15 s)	Portland cement, PAA-containing SBF	Intrafibrillar mineralization within the collagen matrix
Xu *et al.* 2011 [27]	P-chi	Demineralizing solution (72 h)	Remineralizing solution	CaPO_4_ deposited on demineralized dentine
Wang *et al.* 2011 [33]	Peptide	37% PA (15 s)	CaCl_2_ solution, PO_4_ neutralization buffer	Peptide improved remineralization of acid-etched dentine
Zhou *et al.* 2012 [17]	Polydopamine	37% PA (2 min)	CaPO_4_ solution	Polydopamine coating promoted dentin remineralization
Ning *et al.* 2012 [18]	Agarose gel	20% PA (60 s)	CaCl_2_ solution Na_2_HPO_4_ Agarose gel	Apatite completely covered the dentine surface
Qi *et al.* 2012 [24]	PAA, Na_5_P_3_O_10_	pH-cycling	MTA, SBF	MTA effectively promoted dentine remineralization
Zhang *et al.* 2012 [28]	STMP	Demineralizing solution (72 h)	Ca(OH)_2_-treatment, Remineralizing solution	A layer of rod-shaped crystals formed on dentine
Li *et al.* 2013 [20]	PAMAM dendrimer	0.5 M EDTA (30 min), 4 M GuCl	Artificial saliva	Intrafibrillar mineralization process within collagen fibrils
Wang *et al.* 2013 [29]	PAA	37% PA (10 s)	Mineralization solution	Remineralization took place in low but not in high PAA concentration
Cao *et al.* 2013 [31]	STMP	37% PA (60 s)	CPP-ACP, Metastable CaPO_4_ solution	Apatite formation on the phosphorylated collagen fibers
Cao *et al.* 2014 [1]	Oligopeptide	37% PA (60 s)	Metastable CaPO_4_ solution	Apatite completely covered the dentine surface
Osorio *et al.* 2014 [16]	Zn (as bioactive element)	35% PA (15 s)	Artificial saliva	Zn and PO_4_ were crucial for hydroxyapatite homeostasis
Zhou *et al.* 2014 [21]	PAMAM-COOH	0.5 M EDTA (30 min), 4 M GuCl	Artificial saliva	Remineralization of dentine with apatite
Sun *et al.* 2014 [30]	PAA, l-glutamic acid	35% PA (10 s)	Remineralization solution	Dentine remineralization took place
Jia *et al.* 2014 [32]	PAMAM dendrimer	37% PA (10 s)	Artificial saliva	PAMAM promotes mineralization of demineralized dentinal tubules

CPP-ACP—Casein phosphopeptide-amorphous calcium phosphate; EDTA—Ethylenediaminetetraacetic acid; NCPs—Non-collagenous proteins; MTA—mineral trioxide aggregate; PA—phosphoric acid; PAA—Polyacrylic acid; PAMAM—Poly(amidoamine) dendrimer; PBS—Phosphate-buffered saline; P-chi—Phosphorylated chitosan; PVPA—Polyvinylphosphonic acid; SBF—Simulated body fluid; STMP—sodium trimetaphosphate.

The functions of different NCP analogues used in the included articles are clearly presented in Table 2. The most commonly used NCP analogue was Polyacrylic acid (PAA). Five studies added PAA to the phosphate-containing SBF as the sequestration analogues of NCPs [19,22,23,25,26]. Another study used PAA powder mixed with STMP powder and MTA [24]. Two studies added PAA to the calcium phosphate solution [29,30]. The functions of PAA were to simulate calcium phosphate binding sites of DMP1, to stabilize amorphous calcium phosphate as a sequestration agent, to prevent fluidic ACP nanoparticles from aggregating into larger particles, and to transform into apatite prior to their entry into the dentine collagen fibrils.

In addition, a polyphosphate-containing biomimetic analogue such as polyvinylphosphonic acid (PVPA) or sodium trimetaphosphate (STMP) was employed as the template-biomimetic-analogue to bind to the dentine collagen matrix and to further attract ACP nano-precursors into the collagen matrix. Two studies added PVPA and PAA to SBF [23,25]. PVPA acted as the phosphonated template analogue, and PAA acted as the sequestration analogue. Another study added PVPA to demineralized dentine collagen [19]. The functions of PVPA were to simulate collagen-binding function of DMP1, to act as a template-analogue of DMP, inhibitor of matrix metalloproteinases, and to recruit ACP nano-precursors into the collagen matrix. Four studies used STMP solution to demineralize dentine collagen matrix before biomineralization [22,26,28,31]. Another study mixed STMP powder with PAA powder and MTA [24]. The functions of PVPA mentioned in the included articles were phosphorylation of type I collagen, adsorption, and formation of covalent bonds with demineralized collagen matrix. Moreover, it also acts as a template molecule to attract ACP-nanoprecursors to nucleate in the collagen fibrils [19,23,25].

**Table 2:** NCP analogues and their functions in biomimetic mineralization on human dentine.

NCP Analogues	Function of NCP Analogues	Approach [Reference]
Polyacrylic acid (PAA)	● Simulating CaPO_4_binding sites of DMP1	PAA-containing SBF [19,22,26] PAA/PVPA-containing SBF [23] PAA-STMP-MTA [24] PAA/PVPA & PO_4_ solution [25] PAA-CaPO_4_ solution [29] PAA/l-Glu-CaPO_4_ solution [30]
	● Stabilizing ACP	
	● Inhibiting nucleation for ACP stabilization	
	● Prolonging the lifetime of ACP	
Polyvinylphosphonic acid (PVPA)	● Collagen-binding function of DMP1	PVPA-collagen fibril [19] PAA/PVPA-containing SBF [23] PAA/PVPA & PO_4_ solution [25]
	● Templating analogues of DMPs	
	● Inhibiting the activity of MMPs	
	● Recruiting ACP nano-precursors into collagen matrix	
Sodium trimetaphosphate (STMP)	● Phosphorylating of type I collagen	STMP-collagen matrix [22,26,28,31] PAA-STMP-MTA [24]
	● Binding to demineralized collagen matrix	
	● Forming covalent bonds	
	● Attracting ACP-nanoprecursors	
Phosphorylated chitosan (P-chi)	● Binding to collagen	P-chi-collagen matrix [27]
	● Introducing functional groups onto the collagen	
	● Inducing homogenous nucleation	
Peptide	● Binding calcium ions	Peptide-collagen matrix [33]
	● Initiating mineral deposition	
	● Binding collagen by electrostatic interactions	
Agarose gel	● Binding to collagen molecules	Agarose gel-PO_4_-collagen matrix [18]
Polydopamine	● Binding to collagen fiber	Polydopamine-collagen matrix [17]
	● Providing new nucleation site	
Polyamidoamine dendrimer (PAMAM)	● Binding to collagen fibrils	PAMAM-collagen matrix [20,21,32]
	● Recruiting ACP nano-precursors into collagen matrix	
	● Guiding meso-crystals to assemble into large ones	
	● Inducing the periodicity of the mineralized fibrils	
Oligopeptide	● Collagen-binding domain of DMP1	Oligopeptide-collagen matrix [1]
	● Hydrophilic C-terminal of amelogenin	
l-glutamic acid	● Triggering crystallization	PAA/l-Glu-CaPO_4_ solution [30]
	● Promoting calcium phosphate crystallization	
	● Substituting Glu-rich domain of DMP1	

ACP—Amorphous calcium phosphate; BSP—Bone sialoprotein; DMP1—Dentine matrix protein; MMPs—Matrix metalloproteinase.

Apart from the aforementioned NCPs analogues, phosphorylated chitosan (P-chi), polyamidoamine (PAMAM) dendrimers, agarose gel, peptide or oligopeptide, and l-glutamic acid were also used to mimic the functions of NCPs. These NCPs analogues could bind to collagen matrix, and thus induce ACP nano-precursors into collagen matrix.

## 3. Discussion

It is well established that the collagen matrix serves as a scaffold for crystal deposition but does not provide a mechanism for nucleation of hydroxyapatite [34]. The biomineralization process is usually modulated by a series of NCPs, although they only comprise approximately 10% of the organic components [31,35]. In biomineralization of dentine, NCPs with a high affinity for calcium ions and collagen fibrils are responsible for regulating the nucleation and growth of minerals, such as dentine matrix protein (DMP1) and dentine phosphophoryn (DPP, DMP2) with highly phosphorylated serine and threonine residues [36]. These NCPs can induce and regulate biomineralization of dentine *in vivo* by working as nucleator or inhibitor [37]. Therefore, it becomes a general strategy to study the structures and functions of NCPs to biomimetic remineralize the elegant hierarchical structure of dentine. However, it is difficult to extract and purify natural NCPs. Thus, many researchers focus on finding and developing the analogues that can play the role of NCPs in the biomineralization process. Polyacrylic acid (PAA) and polyvinylphosphonic acid (PVPA) were used as the NCP analogues in biomimetic mineralization of dentine [25]. The addition of PAA and PVPA to the Portland cement-PCF/SBF system stabilizes metastable ACP nano-precursors which are small enough to penetrate a demineralized collagen matrix. PAA may simulate the calcium phosphate binding sites of DMP1, and PVPA simulates the collagen-binding function of DMP1 in guiding the nano-precursors’ recruitment to the collagen matrix [25]. Using this dual-analogue biomimetic system, Tay and his colleagues were successful in remineralizing a variety of demineralized dentine matrix with intrafibrillar and interfibrillar remineralization of dentine collagen fibrils [19,23,25]. Based on the aforementioned NCP analogues for biomimetic remineralization of dentine, a series of dual-analogue biomimetic systems, *i.e.*, the combination of polycarboxylic acid (such as PAA) as the ACP stabilization analogue and phosphate-based template-analogue (such as PVPA or STMP), has been developed to mimic the full role of NCPs and resulted in highly ordered intrafibrillar nano-apatite assembly [22,24,26,30,34]. STMP, which has been frequently used in the food industry as a chemical phosphorylating reagent, can absorb type I collagen via an electrostatic mechanism and a chemical phosphorylation mechanism [28,38,39]. The phosphorylated dentine collagen matrix functions as a template-molecule to attract ACP nano-precursors and to nucleate apatite within the collagen fibrils resulting in the formation of intrafibrillar and interfibrillar remineralization of dentine [22]. A study compared the effects of phosphorylated and non-phosphorylated dentine collagen matrix in formation of intrafibrillar remineralization of dentine [31]. However, in the presence of ACP nano-precursors, the non-phosphorylated dentine collagen matrix could not form the intrafibrillar remineralization of dentine. Apart from the PVPA and STMP, phosphorylated chitosan [27], peptide/oligopeptide [3,33], and PAMAM dendrimer [20,21,32] also functioned as the template-analogues for the biomimetic remineralization of dentine collagen matrix.

Type I collagen accounts for about 90% of the organic matrix of dentine. As the caries progresses into dentine, the acid produced by bacteria results in the exposure of type I collagen. Recent studies indicated that type I collagen plays an active role in the biomineralization process by acting as templates for attraction of ACP nano-precursors. In this review, different methods were used to expose the type I collagen or create the carious-like lesions. Phosphoric acid at 37% was the most commonly used method in the studies, and this acid-etching protocol is also commonly employed in clinical dentistry. A study indicated that acid-etching mineralized dentine with 37% PA for 15 s could create a 5 µm-thick layer of mineral-free collagen matrix [25]. Similar results were also obtained by the other researchers, such as etching dentine surface with 32% PA for 15 s to create a 5–8 µm-thick layer of completely demineralized collagen matrix [26], 37% PA for 10 s to create a 2–4 µm-thick layer of mineral-free collagen matrix [29], and 35% PA for 10 s to produce a 3–4 µm-thick artificial demineralized dentine layer [30]. Different concentrations (20%–37%) of PA were also used to expose the dentine collagen matrix [1,17,18,31,32,33]. It is suggested that treatment of dentine with 37% PA for less than 1 min does not denature the dentine collagen matrix [40,41].

Some researchers are concerned about the potential denaturation of PA on dentine collagen matrix [41]. The EDTA-etching approach became an alternative to remove minerals. EDTA treatment could maintain an intact dentine collagen matrix and provided a mineral-free layer near the dentine surface [20]. In this review, some researchers used GuCl to remove NCPs on the EDTA-demineralized dentine surface [20,21,26]. This protocol is especially suitable to study the NCP analogues on the biomimetic remineralization of dentine. Apart from PA and EDTA treatments, the pH-cycling procedure was used to create a partially demineralized dentine. It could mimic the dynamic variations on mineral saturation in the natural caries process [42,43].

Silva [44] classified the synthesis of biomaterials as top-down or bottom-up approaches. The top-down approach begins with a bulk material that incorporates critical nanoscale details. Classical ion-based mineralization strategies often involve the use of metastable calcium and phosphate ion-containing solutions and gels [45]. This example of a top-down mineralization approach occurs by epitaxial growth over existing seed crystallites, which cannot occur by spontaneous nucleation of minerals on the organic matrix, such as demineralized dentine. In the absence of biomimetic analogues of NCPs, there should be no intrafibrillar apatite deposition using the top-down mineralization approach. In this review, some of the results only showed interfibrillar apatite deposition and occlusion of dentinal tubules, such as using bioactive materials [12,13,14], agarose gel system [18], and zinc [16]. Although two research groups successfully synthesized peptide [33] or oligopeptide [1] to partly mimic the function of NCPs, they did not reproduce the structural hierarchy of intrafibrillar apatite deposition within the collagen matrix due to the lack of ACP nanoparticle formation. Even so, these results are clinically significant for the management of dentine hypersensitivity.

The mineral phase in dentine can be classified as intrafibrillar mineral, which is confined within or immediately adjacent to the gap zones of the collagen fibrils; and interfibrillar mineral, which locates within the interstitial spaces separating the fibrils [46]. After demineralization, the interfibrillar mineral of dentine is rapidly dissolved. The intrafibrillar mineral is partially or fully dissolved and the gap zones become visible. Intrafibrillar mineral plays an important role in the mechanical properties of dentine [47]. Intrafibrillar remineralization is confined that the mineral grows and fills into the gap zones of the collagen fibrils. In contrast to the top-down approach, the bottom-up approach starts with one or more defined molecular species, which undergoes certain processes that result in a higher-ordered and organized structure, such as self-assembly of amorphous nano-precursor particles and their subsequent mesoscopic transformation in biomineralization [48]. This non-classical particle-mediated crystallization pathway involves a multistage process [49]. The calcium and phosphate ions self-assemble into prenucleation clusters. In the presence of NCP analogues, these prenucleation clusters aggregate into amorphous ACP nano-precursors. Then these precursors penetrate into the gap zones of collagen fibrils and further grow into apatite along the intrafibrillar space of collagen. The intrafibrillar remineralization of collagen fibrils results in interfibrillar remineralization between adjacent collagen fibrils. Therefore, formation of amorphous ACP nano-precursors is the fundamental step in biomimetic remineralization of dentine.

Although the biomimetic remineralization methods published in the literature have potential in remineralizing carious dentine, these techniques were performed in the laboratory and remained a proof-of-concept. The models are also simplistic, which is very different from the *in vivo* environment. It is clinically relevant to examine if the biomimetic remineralization protocol is applicable to *in vivo* dentine carious lesions. Many studies did not account for the pellicle and biofilm, which can prevent the biomimetic remineralization. There are challenges, and it is still a long way before the strategies can be used in clinical care to benefit patients. Non-caries tooth loss is caused by attack of non-bacterial acid. The process of dental caries is more complicated because of the involvement of bacteria. The outer caries-infected dentine contains aciduric bacteria and its metabolic products, making carious lesions difficult to be remineralized. The next step may involve designs for *in situ* applied delivery system for the ACP-stabilization analogues with anti-bacterial property. It is plausible for biomimetic remineralization of dentine to be accomplished through these bio-agents to be incorporated into restorative materials.

## 4. Methods

### 4.1. Search Strategy

A systematic search of the publications in the PubMed, TRIP, and Web of Science databases was performed to identify manuscripts that could be included. Search words are as follows: ((biomimetic and (mineralization or mineralisation or regeneration or remineralization or remineralisation)) or biomineralization or biomineralisation) and (dentin or dentine). Regarding computer searches of databases, no publication year or language limit was used, and the last search was made on 1 January 2015.

### 4.2. Screening Methods and Data Extraction

In the first stage, titles and abstracts of initially identified articles were screened independently by two authors (Chris Ying Cao and May Lei Mei) (Figure 1). They selected for further review if the articles met the inclusion criteria. Inclusion criteria for the studies were the use of biomimetic mineralization methods for demineralized human dentine in laboratory tests. Clinical trials, reviews, non-English articles, animal teeth, resin-dentine interface studies, hybrid layer studies, hybrid scaffolds studies, and irrelevant studies were excluded. Duplicated articles were eliminated. If consensus were not reached, the article would be discussed with the third author (Quan-Li Li) for final decision. In the second stage, the remaining articles were retrieved with full texts. Manual screening was conducted by the two authors (Chris Ying Cao and May Lei Mei) on the references lists of all included articles to identify relevant articles that could fulfill the inclusion criteria, and the full texts of potentially interesting studies were examined. Disagreements about the inclusion or exclusion of a study were resolved after discussion with a third author (Quan-Li Li). A protocol for data extraction was defined and evaluated by two authors (Chris Ying Cao and May Lei Mei). Data were extracted from full-text articles by one author (Chris Ying Cao) and reviewed by another author (May Lei Mei). Similar information was categorized into groups such as NCP analogues and summarized the functions of the material and methods mentioned in the included articles.

## 5. Conclusions

In conclusion, a number of studies reported *in vitro* success in biomimetic mineralization of dentine with different methods, including the use of NCP analogues and using bioactive materials. The use of NCP analogues was successful in remineralizing a variety of demineralized dentine matrix with intrafibrillar and interfibrillar remineralization of dentine collagen fibrils. These models are chemical models with no bacterial involvement and are different from *in vivo* conditions.
